# Production of BP178, a derivative of the synthetic antibacterial peptide BP100, in the rice seed endosperm

**DOI:** 10.1186/s12870-017-1011-9

**Published:** 2017-03-14

**Authors:** Laura Montesinos, Mireia Bundó, Esther Badosa, Blanca San Segundo, María Coca, Emilio Montesinos

**Affiliations:** 1 Institute of Food and Agricultural Technology-CIDSAV-XaRTA, University of Girona, Girona, 17071 Spain; 2 Centre for Research in Agricultural Genomics (CRAG), CSIC-IRTA-UAB-UB. Edifici CRAG, Campus de la UAB, 08193 Bellaterra, Barcelona Spain

**Keywords:** Antimicrobial peptide, *Oryza sativa*, Rice biofactory, Protein bodies, Peptide recovery, Pathogen resistance

## Abstract

**Background:**

BP178 peptide is a synthetic BP100-magainin derivative possessing strong inhibitory activity against plant pathogenic bacteria, offering a great potential for future applications in plant protection and other fields. Here we report the production and recovery of a bioactive BP178 peptide using rice seeds as biofactories.

**Results:**

A synthetic gene encoding the BP178 peptide was prepared and introduced in rice plants. The gene was efficiently expressed in transgenic rice under the control of an endosperm-specific promoter. Among the three endosperm-specific rice promoters (*Glutelin B1*, *Glutelin B4* or *Globulin 1*), best results were obtained when using the *Globulin 1* promoter. The BP178 peptide accumulated in the seed endosperm and was easily recovered from rice seeds using a simple procedure with a yield of 21 μg/g. The transgene was stably inherited for at least three generations, and peptide accumulation remained stable during long term storage of transgenic seeds. The purified peptide showed in vitro activity against the bacterial plant pathogen *Dickeya* sp., the causal agent of the dark brown sheath rot of rice. Seedlings of transgenic events showed enhanced resistance to the fungal pathogen *Fusarium verticillioides*, supporting that the *in planta* produced peptide was biologically active.

**Conclusions:**

The strategy developed in this work for the sustainable production of BP178 peptide using rice seeds as biofactories represents a promising system for future production of peptides for plant protection and possibly in other fields.

**Electronic supplementary material:**

The online version of this article (doi:10.1186/s12870-017-1011-9) contains supplementary material, which is available to authorized users.

## Background

Antimicrobial peptides (AMPs) are components of the innate immune system in animals and plants, and play an important role in antagonistic relationships between microorganisms [1–5]. There is a great expectation in biotechnological applications of AMPs that include their use as therapeutic agents, biopreservatives in cosmetics, materials and food; and substitutes of antibiotics in animal feed and in crop protection. AMPs are of particular interest in the field of crop protection because they meet the regulatory requirements for low environmental impact of pesticides [3]. However, the exploitation of natural AMPs presents limitations, as they are produced in low amounts in the living organisms and their extraction and purification requires complex and costly procedures. In addition, natural AMPs might show toxicity against non-target organisms [6].

The rational design of AMPs may overcome these limitations by developing peptides with improved biological properties, like increased antimicrobial activity and stability, and reduced toxicity against non-targeted organisms [7–9]. In this context, a library of synthetic linear undecapeptides consisting of cecropin A (1–7)-melittin (2–9) hybrids (named CECMEL11 library), was previously obtained by combinatorial chemistry [8]. Several peptides from this library showed potent antibacterial and/or antifungal activities with low cytotoxicity (hemolytic and phytotoxic activity) and moderate susceptibility to proteolytic degradation [6]. Among them, the BP100 peptide exhibited strong activity against phytopathogenic bacteria and was effective in controlling infections by different phytopathogens in apple, pear, tomato and pepper [7]. Another set of CECMEL11 peptides (e.g. BP15, BP21) showed fungicidal activity and were effective in the control of postharvest rot of fruits [10] and brown spot of pear trees [11]. Some of the CECMEL11 peptides (e.g. BP100, BP16) had cell penetrating properties in eukaryotic cells and have already been used to deliver conjugated “cargo” compounds intracellularly [12, 13]. Thus, CECMEL11 peptides represent useful tools in different fields of application. However, their exploitation requires the development of sustainable and feasible means of production.

Production of AMPs in living systems used as biofactories appears to be a promising alternative to the chemical synthesis [14]. Several prokaryotic and eukaryotic expression systems for the production of heterologous proteins or peptides have been described using microbial, insect or mammalian cells, and plants. All have advantages and disadvantages, depending on the type of protein to be produced, the complexity of downstream processes to recover it, and the cost. Microbial systems based on bacteria and yeasts, provide high yield production by fermentation processes and are easy to handle, but the inclusion body formation requires solubilization and refolding [15–17]. Procedures and the use of insect and mammalian cells, or transgenic animals are complex and expensive, with the additional disadvantages of the slow-scaling up and the ethical concerns [18]. Transgenic plants offer several advantages for production of AMPs in a cost effective manner, and therapeutic proteins have already been produced [19–28]. In recent years, commercial plant-derived proteins have been produced using cereal seeds [27, 29–32], and rice has been chosen in several cases as a platform [23, 33, 34]. Different rice promoters showing a seed-specific expression pattern have been described [35, 36], and valuable recombinant proteins including vaccines, antibodies, cytokines, hormones or enzymes have been successfully produced in rice seeds [31, 37–42]. Most of these rice based platforms produced peptides as multimers [43–45] or as a fusion to a large protein carrier such as storage proteins [46–50]. However, several reports show the difficulty to produce small peptides in the rice seed due to its small size, low stability and/or susceptibility to protease degradation in planta [44, 47, 49, 51]. In order to achieve the production of CECMEL11 peptides using plant biofactories, synthetic analogs of the BP100 peptide that fulfill specific requirements for optimal production in plants were previously described [52]. Peptide modifications were made to increase peptide size, and to incorporate retention signals targeting the peptide to specific cell compartments (protein bodies, PB). *A priori* this strategy reduces the risks of AMP degradation and/or toxicity towards plant cells. The compounds (e.g. BP178) were evaluated for antimicrobial, hemolytic and cytotoxic activities, and several were considered as suitable candidates to be produced in plant biofactories.

The aim of the present study was to determine the feasibility of using the rice seed as a biofactory to produce and recover a BP100 derivative, the BP178 antibacterial peptide (29 amino acids, MW = 3.2 kDa). This peptide has a lytic mode of action on several plant pathogenic bacteria. Promoters of rice seed storage protein genes were used to direct endosperm-specific expression of the *AMP* gene, and stable transgenic rice plants accumulating the AMP in the seed endosperm were generated. The peptide was easily purified from rice seeds, and showed antimicrobial activity. Moreover, transgenic seedlings showed resistance to pathogen infection suggesting that the plant produced BP178 peptide was also biologically active in the rice seed.

## Results

Based on the library of BP134 derivatives [52], in which modifications in peptide sequences were introduced to facilitate their production in plant systems, we selected the BP178 peptide for its production in rice seeds. This peptide consisted of a magainin fragment linked to the original sequence of the BP134 through an AGPA hinge region, and the KDEL retention signal sequence (KKLFKKILKYLAGPAGIGKFLHSAK-KDEL-OH) at the C-terminus (Additional file 1). We reasoned that retention into the endoplasmatic reticulum will protect the peptide from proteolytic degradation while reducing the risk of toxicity to the plant cell. The BP178 peptide exhibited strong bactericidal effect against several plant pathogenic bacteria with very low hemolytic activity. Phytotoxicity of BP178 to tobacco cells was observed by infiltration only at concentrations 50–100 times higher than minimal inhibitory concentration MIC values [52]. The selected peptide has a strong cationic net charge (pI = 10.84) with an amphipathic character (Additional file 2).

### Generation and characterization of transgenic rice plants expressing a synthetic *BP178* gene in seed tissues

**Fig. 1:**
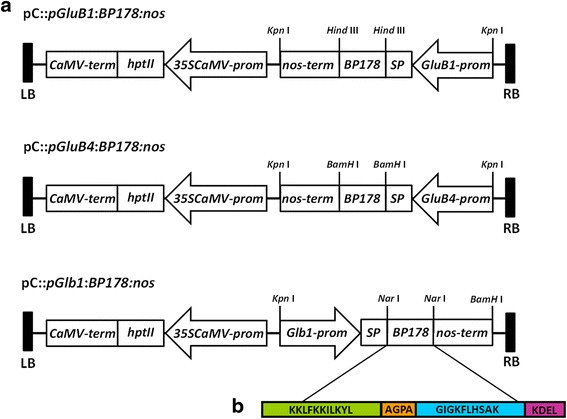
Schematic representation of the plant expression vectors for the expression of the *BP178* gene in the rice endosperm. **a** Three different constructs in pCAMBIA vector (pC) in which the synthetic gene was cloned between the endosperm-specific promoter including the signal peptide coding sequence of the corresponding seed storage protein (SP) and the *nopaline synthase* terminator (*nos*). Relevant restriction enzyme sites for cloning purposes are indicated. The *hptII* gene encoding resistance to hygromycin under the control of the CaMV35S promoter and terminator was contained into the T-DNA region of pC. LB, left border; RB, right border of T-DNA. Arrows indicate the orientation of the sequence. **b** The chimeric BP178 peptide corresponding to the fusion of BP134 (KKLFKKILKYL-OH, a cecropin A (1–7)-melittin (2–9) hybrid, in *green*) linked through the hinge sequence (AGPA, in *orange*) to a magainin fragment peptide (GIGKFLHSAKKFGKAFVGEIMNS –OH, in *blue*); and extended with ER retention signal (KDEL, in *pink*). For details, see Additional file 1: Figure S1

Three different constructs were obtained in which the synthetic *BP178* gene was cloned under the control of an endosperm-specific rice promoter (i.e. *pGluB1:BP178:nos*, *pGluB4:BP178:nos* and *pGlb1:BP178:nos*) (Fig. 1). The promoters and the signal peptide coding sequence of the rice *glutelin B1*, *B4* and *globulin* genes were used to direct the expression of the *BP178* gene and accumulation of the transgene product in the seed endosperm.

Transgenic T0 and homozygous plants were morphologically similar to the wild-type untransformed plants.

All the regenerated rice lines recovered from different transformation events had the complete cassette for *BP178* expression integrated into their genomes, as revealed by PCR analysis and subsequent sequencing of the PCR products (Additional file 3). Most transgenic lines carried a single copy insertion of both selectable marker and the *BP178* gene, that is 66.0% for the *pGlb1:BP178*-lines, 100% for the *pGluB4:BP178*-lines and 50% for the *pGluB1:BP178*-lines. As expected, a 3:1 segregation ratio was observed in T0 events harbouring a single copy of the transgene. Single copy events were used as the parental lines to obtain homozygous lines in the T2 generation. Thus, independent homozygous lines from *pCGlb1:BP178:nos* (5 lines), *pCGluB4:BP178:nos* (3 lines) and *pCGluB1:BP178:nos* (3 lines) transformation events were selected for further analysis. Additionally, qPCR analysis confirmed stable transgene inheritance through successive generations (at least up to the T4 generation).

### Production and accumulation of BP178 peptide in transgenic rice seeds

**Fig. 2:**
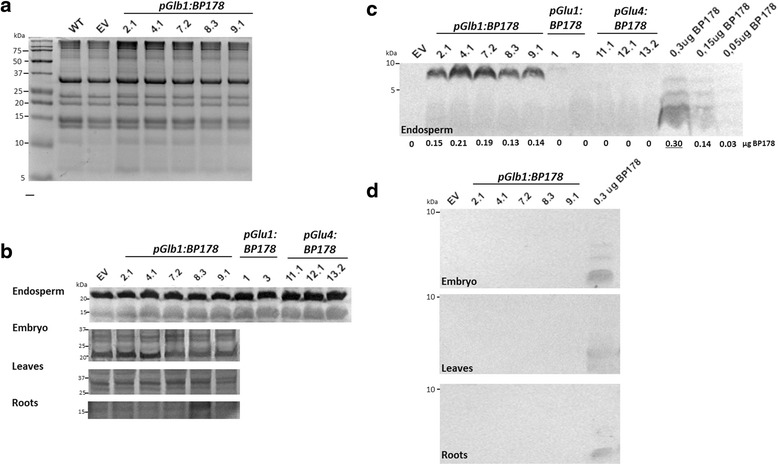
BP178 peptide accumulates in the endosperm of *Glb1:BP178* transgenic rice seeds without altering accumulation levels of native storage proteins. **a** Coomassie Blue staining of PB extracts from the indicated seeds. EV indicates seeds from plants transformed with the pCAMBIA 1300 empty vector. **b** Ponceau staining of protein samples (50 μg) **c** Western-blot analysis and quantification of the BP178 peptide in the protein body-enriched fractions (50 μg) from mature seed endosperms of the indicated *pGlb1:BP178*, *pGluB4:BP178*, *pGluB1:BP178* and empty vector (EV) lines. The accumulation levels of BP178 peptide were quantified with the Quantity tools of the Chemidoc program using the 0.3, 0.15 and 0.05 μg amounts of synthetic BP178 peptide as a reference (taking into account the sum of the three bands observed). Numbers below the western blot lanes correspond to μg of BP178 peptide. **d** Western-blot analysis of protein body-enriched fractions (50 μg) from embryos, leaves or roots of the indicated *pGlb1:BP178*, and empty vector (EV) lines. For comparative purposes, 0.30, 0.15 and 0.05 μg of synthetic BP178 peptide were run simultaneously in the Tricine-SDS gels. Immunodetection was performed using specific polyclonal anti-BP178KDEL antibodies and coloured phosphatase alkaline reaction

The total protein profiles of transgenic and WT seeds were initially examined by SDS-PAGE, and no significant differences were observed in the accumulation of native storage proteins (e.g. glutelins, prolamins) when comparing transgenic and WT seed protein profiles (Fig. 2a and b).

The promoters and the signal peptide coding sequence of the rice *glutelin B1*, *B4* and *globulin* genes were used to direct the expression of the *BP178* gene and accumulation of the transgene product. Previous studies demonstrated the capability of these regulatory sequences to produce the cecropin A peptide in the protein bodies of the rice endosperm. Accordingly, in this work we prepared protein body (PB)-enriched fractions from mature seeds of transgenic lines harbouring a BP178 construct following the procedure previously described [54] (Additional file 4).

Western-blot analysis of PB proteins, using an anti-BP178 polyclonal antibody, revealed accumulation of an immunoreactive band, putatively BP178 peptide, in the PB enriched fraction of the transgenic seeds harbouring the *pGlb1:BP178* construct, which was absent in the same fraction of the empty vector seeds (Fig. 2c). Also, a large variation in accumulation of BP178 was observed among the different *pGlb1:BP178* lines generated (Additional file 5). We also noticed that the apparent molecular weight of the BP178 peptide in Tricine-SDS PAGE was larger (~8 kDa) than the expected molecular weight for the corresponding peptide (3.2 kDa). In addition, when high concentrations of the synthetic BP178 peptide were subjected to immunoblot analysis, at least three immunoreactive bands of different mobility were detected (see Fig. 2), that could be associated to peptide multimerization. No accumulation of the BP178-immunoreactive band was observed in protein extracts obtained from embryos, leaves or roots from plants that expressed the *BP178* gene under the control of the seed-specific promoter (Fig. 2d).

**Fig. 3:**
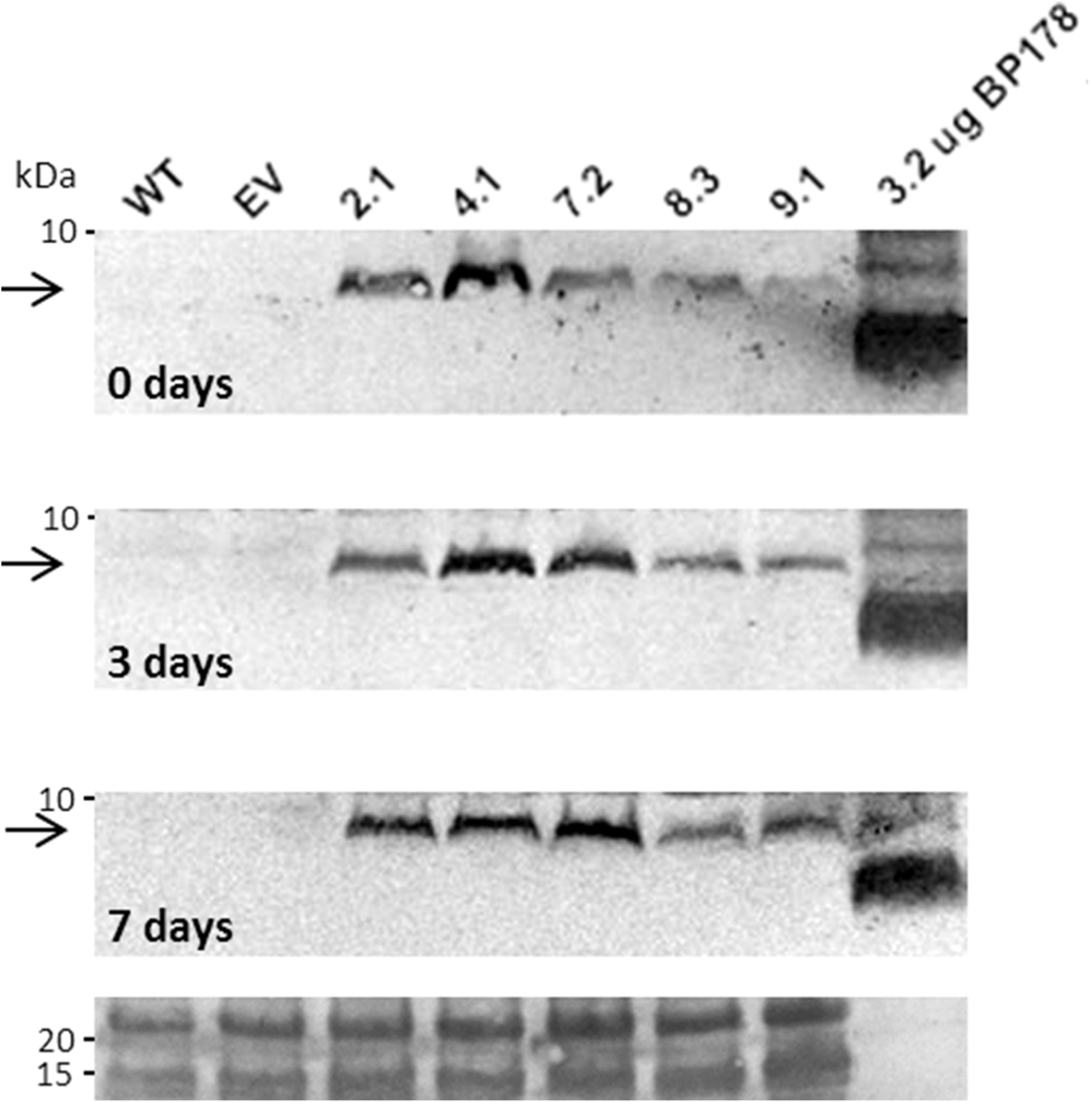
Stability of the BP178 peptide accumulated in 3 years-old seeds. The endosperm tissue was manually dissected from mature seeds (0-days) and germinating rice seeds (3 and 7 days of germination). Protein extracts (35 μg) were separated by Tricine-SDS gels, transferred onto PVDF membrane, and incubated with the anti-BP178 antibody followed by anti-rabbit IgG (Fc) alkaline phosphatase conjugated secondary antibody. EV, rice plants with the empty pCAMBIA 1300 vector were used as a negative control, and 3.2 μg of synthetic BP178 were used as reference control. Arrows, indicate BP178 A-related bands in rice extracts. Numbers in the left side indicate the size of the molecular markers (kDa). Lower panels show Ponceau staining of protein samples

LC-MS/MS proteomic analyses allowed the detection of tryptic-derived peptides from BP178 that were unequivocally identified in the BP178-enriched protein body fraction of *pGlb1:BP178* lines, but not in the empty vector controls. The expected tryptic fragments for the peptide are indicated in Additional file 1 (highlighted sequences). LC-MS/MS data also confirmed that the ~8 kDa immunodetected polypeptide (Figs. 2c and 3; Additional file 4) corresponded to the expected BP178 peptide in seeds of the *pGlb1:BP178* lines, whereas the tryptic peptides from the Glb1 signal peptide were not detected in the PB samples. Analysis by LC-MS/MS also demonstrated that BP178 accumulated in the protein body enriched fraction obtained from *pGluB1:BP178* and *pGluB4:BP178* seeds, but at very low levels (~3 fmols/μL). Among the three strategies assessed for AMP production, the *pGlb1:BP178* seeds showed the highest levels of BP178 accumulation. Among other factors, the orientation of the expression cassettes could be associated with the increased expression of the BP178 gene in the *pGlb1:BP178* compared to that of *pGluB1:BP178* or *pGluB4:BP178* seeds.

Further analysis confirmed that the BP178 peptide remained stable during the germination process, as the peptide was detected in the endosperm tissue of the germinating seeds (3 and 7-day germinated seeds). No BP178-related degradation products were observed in protein extracts from germinating seeds (Fig. 3). Moreover, the *in planta* produced BP178 peptide was found to accumulate in a stable manner during storage of the rice seeds as it could be detected in the transgenic seeds even after long periods of storage (up to 3 years), when stored at room temperature.

### Preparative scale purification and yield estimation of the BP178 peptide produced in seeds of transgenic rice

**Fig. 4:**
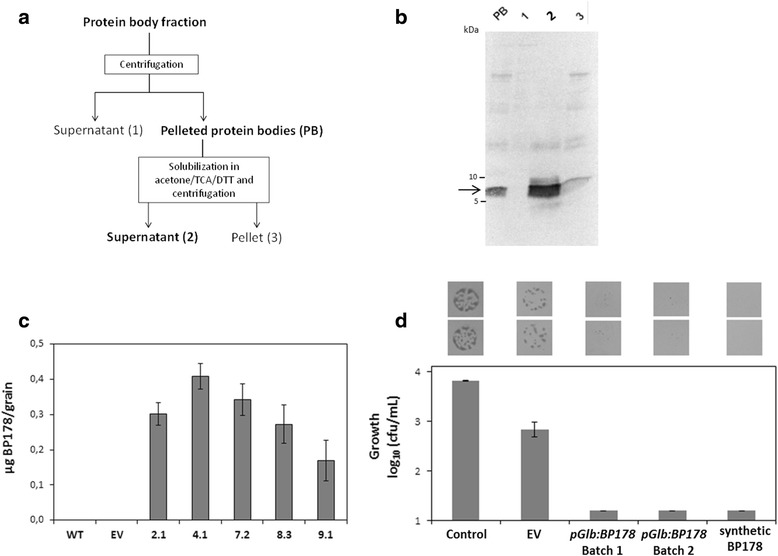
Purification, quantification and antibacterial activity of the BP178 peptide recovered from the *pGlb1:BP178* rice seeds. **a** Schematic procedure for purification. BP178 peptide was recovered at high purity in the acetone supernatant (2) **b** Tris-Tricine SDS-PAGE and western-blot analysis using anti-BP178 antibodies of fractions recovered during the purification process described in (**a**) starting from protein body fractions (50 μg) from seeds from *pGlb1:BP178* line 7.2. Numbers in the top of panel (**b**) correspond to: 1, Supernatant; 2, Pelleted protein bodies; 3, Pellet. **c** Peptide yield from seeds of the indicated *pGlb1:BP178* events*.* The amount of BP178 was calculated by comparing the signal intensity of known amounts of standard (synthetic peptide). Values correspond to the mean of at least 3 independent assays and the standard error is indicated. **d** Antimicrobial activity against *Dickeya* sp. of two batches of purified BP178-fractions from *pGlb1:BP178* lines. Empty vector (EV) and reference synthetic BP178 peptide were used as control. Mean values of two replicates and the standard error are indicated

Knowing that the BP178 peptide accumulates in the rice endosperm being detected in the PB enriched fraction, we focused on the development of a method suitable for purification of this peptide from transgenic rice seeds at a preparative scale. The transgenic line 7, harbouring the *pGlb1:BP178* gene construct presented the highest level of resistance to *Dickeya* sp. infection (see below). For this, transgenic plants harbouring the *pGlb1:BP178* (line 7) were selected. PB enriched fractions were obtained from 1.500 seeds (30 g) of transgenic *pGlb1:BP178* event. As control, PB fractions were obtained from empty vector plants. The procedure used for purification of BP178 from rice seeds is shown in Fig. 4a. In each case, approximately 250 mg of PB proteins were obtained. Next, protein extracts were obtained from PB enriched fraction by precipitation using acetone-TCA-DTT. Interestingly, under these conditions, the BP178 was found to be soluble, and the precipitated seed storage proteins were easily removed from the protein body extracts by centrifugation. BP178 peptide was recovered from the acetone fraction, processed as described in the experimental procedures, and further used. Western blot analysis confirmed the presence of BP178 peptide in the acetone fraction (Fig. 4b). This procedure was used to recover the BP178 peptide accumulating in seeds of five independent *pGlb1:BP178* lines. The BP178 lines accumulated the peptide in the range of 0.17 to 0.41 μg /grain, as determined by western-blot analysis using dilution series of BP178 (Fig. 4c). The highest contents of BP178 was found in rice seeds of line 4.1, as 0.410 ± 0.037 μg/grain (21 μg/g of seed, 6.33 pmols/mg of seed). The mean BP178 peptide yield for the 5 homozygous BP178 lines was 0.299 ± 0.039 μg BP178/grain for the 3 year-old seeds, and 0.252 ± 0.085 μg BP178/grain in the case of fresh seeds (0 year-old seeds). There were no statistically significant differences between the BP178 accumulation levels among 0 year-old and 3 year-old seeds, according to an ANOVA analysis (*F* = 0.251; *P* = 0.630).

### BP178 peptide produced in rice seeds exhibits biological activity

The antimicrobial activity of the *in planta* produced BP178 peptide was assessed against the seedling bacterial pathogen *Dickeya* sp. using a contact test. The bacterial suspension was incubated with BP178 purified from *pGlb1:BP178* seeds or with purified fractions from empty vector seeds. The BP178 contents of the two batches of plant derived BP178 used were 0.41 and 0.46 μg/μl, respectively. As expected, empty vector PB enriched fractions had no BP178. Therefore, in the antimicrobial activity assay, the corresponding total amounts were for the synthetic BP178 of 0.05 μg, and for the purified BP178 from transgenic seeds of 2.12 and 2.28 μg, for each batch, respectively. The plant derived BP178 extracts and the reference synthetic BP178 peptide gave similar bactericidal activities (Fig. 4d), but the concentrations of the plant derived BP178 peptide were around forty times higher.

### Enhanced resistance of BP178-rice seeds to infection by bacterial and fungal plant pathogens

**Fig. 5:**
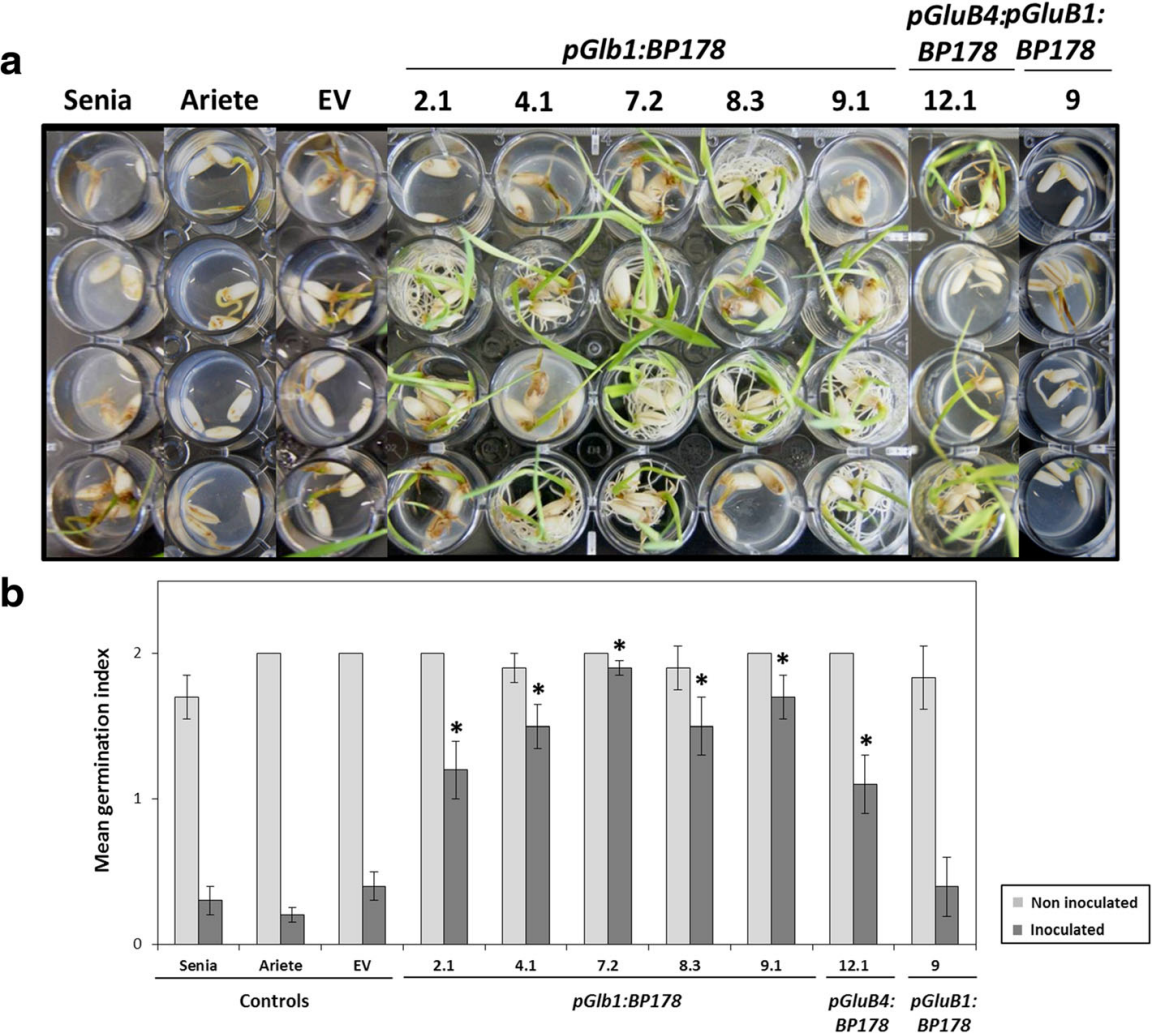
Resistance of BP178 rice seeds to *Dickeya* sp. infection. **a** Phenotypical appearance of wild-type (Senia and Ariete cultivars), empty vector (EV) and the transgenic seedlings carrying the indicated transgenes after 7 days in contact with *Dickeya* sp. 1552.10.1 bacterial suspensions (10^7^ cfu/mL). Pictures are representative of at least 2 independent assays in which 12 seeds per line were analysed. **b** Germination capability of *Dickeya*-infected T3 rice seeds expressing the *BP178* gene. Mean germination index (12 seeds) in inoculated (dark grey) or non-inoculated (pale grey) seeds at 7 days after inoculation. Values correspond to the mean germination index of two independent assays. The standard error is also indicated. The asterisks indicate statistically significant differences compared to the WT and empty vector mean (Tukey’s test, *p < 0.001)

Because the BP178 was found to accumulate in the rice seeds, we investigated resistance to pathogen infection in germinating seeds and seedlings. *Dickeya* sp. 1552-10.1, a seedling bacterial pathogen, was used (Fig. 5). In the presence of *Dickeya* sp., seedlings expressing *pGlb1:BP178* and *pGluB4:BP178* were able to germinate and presented normal root and shoot morphology comparable to that of control seedlings. These seedlings, showed a consistent phenotype of resistance to infection compared to control plants (WT, empty vector) that showed susceptibility to *Dickeya* infection. Differences in susceptibility occurred among lines harbouring the same transgene. In contrast, the *pGluB1: BP178* lines showed susceptibility to *Dickeya* sp., at similar levels than control seeds. These plants exhibited short and brownish shoots, and in most cases were unable to germinate in the presence of the bacterial phytopathogen.

**Fig. 6:**
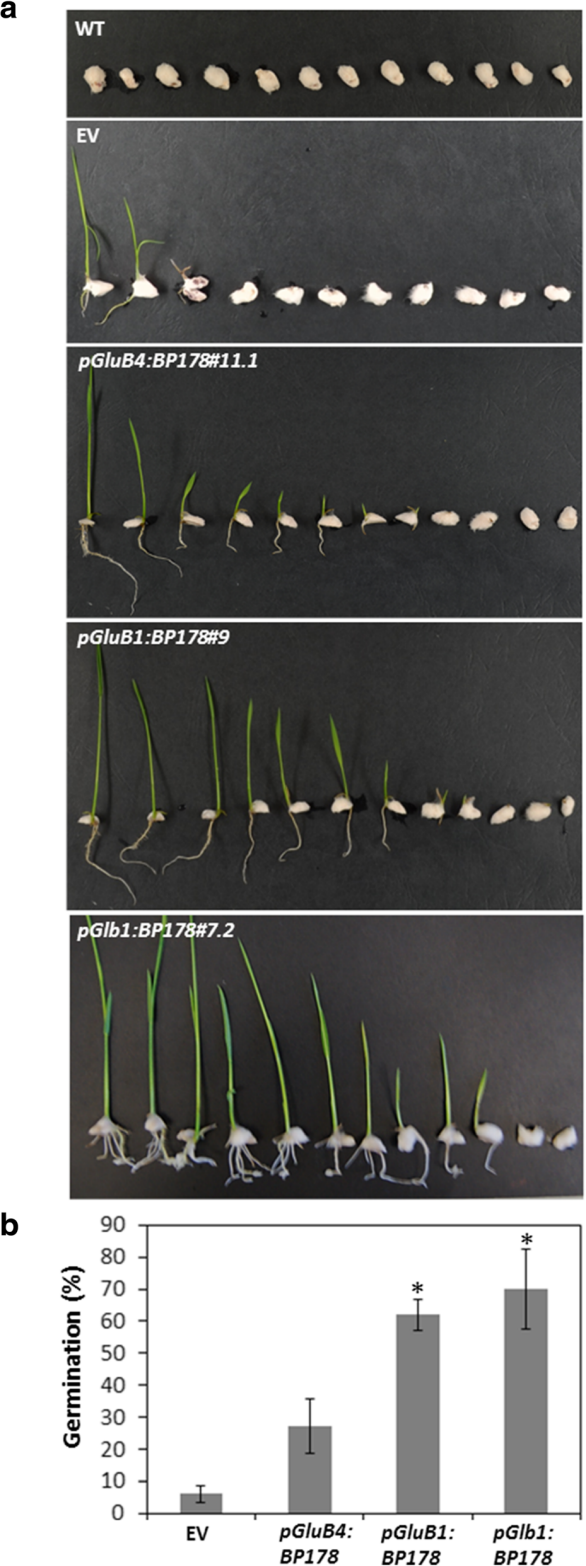
Resistance of BP178-rice seeds to *Fusarium verticillioides* infection. **a** Phenotypical appearance of wild-type (WT) and transgenic seedlings carrying the indicated transgenes or the empty vector (EV) 7 days after germination in contact with *F. verticillioides* spore suspensions (10^5^ spores/mL). Pictures are representative of 3 independent assays **b** Germination capability of the fungal inoculated transgenic seeds referred to wild-type seeds. Values correspond to the mean germination rate and standard errors of three independent assays in which at least 3 independent lines per transgene were analysed. Asterisks denote statistically significant differences with wild-type and empty vector plants (Tukey’s test, *p < 0.001)

Resistance of BP178-rice seedlings against *F. verticillioides*, a seed-borne fungal pathogen associated with Bakanae in rice, was also assessed. It was observed a better germination capability of the fungal-infected transgenic seeds expressing the *BP178* gene under the control of an endosperm-specific promoter, compared to control seeds (WT, empty vector) (Fig. 6). Most of WT and EV seeds were unable to germinate in the presence of the fungal pathogen and in some cases infected seedlings also show crown and stem rot, as well as low number of roots. More in detail, the expression of a BP178 gene under the control of the *GluB1* and *Glb1* promoter resulted in higher level of protection to *F. verticillioides* infection than *GluB4:BP178*-seeds.

## Discussion

In this work, we generated transgenic rice plants accumulating the BP178 peptide in the rice seed endosperm, using endosperm-specific promoters to drive transgene expression. The DNA sequence encoding the N-terminal signal peptide of rice storage proteins was fused to the *BP178* gene, which also contained a C-terminal extension encoding the KDEL sequence for retention in the ER. The homozygous rice seeds accumulating the BP178 peptide showed resistance to infection by two plant pathogens, suggesting that the *in planta*-produced peptide was biologically active. Moreover, the BP178 peptide was easily purified from the transgenic rice seeds and was biologically active. There are several lines of evidence to suggest that BP178 accumulates in PBs, while subcellular location was not confirmed by direct methods (e.g. immunolocalization). Such evidences include the accumulation of a similar protein peptide (Cec A) to PBs when identical regulatory sequences were utilized for cloning and transformation, and also the presence of BP178 in cell fractions that have been shown to be enriched for protein bodies [53, 54].

It is worthwhile to mention that previous studies failed to obtain stable transgenic rice plants expressing certain *BP100* derivative genes under the control of a constitutive promoter, suggesting toxicity of these AMPs to the host plant [55, 56]. In a few cases, the *BP100* derivative genes were constitutively expressed in rice, but resulted in abnormal development of the plants [55]. On the other hand, the suitability of endosperm-specific promoters to drive expression of the *cecropin A* gene in the rice endosperm has been demonstrated [54]. Altogether, these findings highlight the relevance of using the appropriate strategy for production of AMPs in transgenic rice to avoid undesirable effects, particularly for production of the BP178 antibacterial peptide in rice seed tissues.

The BP178 peptide accumulated in the endosperm tissue of the transgenic seeds, but not in embryo, leaf or root tissues, which is in agreement with the already described seed-specific activity of the *GluB1*, *GluB4* and *Glb1* promoters [35]. It is known that both glutelin and globulin rice seed storage proteins are synthesized in the endoplasmic reticulum and deposited in the PBII (also called protein storage vacuoles, [57]) of rice endosperm, due to cis-acting RNA localization signals present in the transcripts [58]. However, the sorting signals for seed storage proteins deposition into PBII have not yet been identified in rice [59]. Our results illustrate the utility of using an endosperm-specific promoter and signal peptide sequence of seed storage proteins for production of BP178 in protein body enriched fractions of transgenic rice seed. In this context, there are examples with other peptides in which a similar strategy has been successful [54] as well as unsuccessful [60]. Therefore, it should be considered the possibility that the BP178 peptide accumulates without the targeting signal sequence in the signal peptide. For instance, the BP178 peptide might interact with seed storage proteins, as has been previously described for other peptides [40, 61]. Even, protein aggregation may also play a role in protein sorting, as it has been reported for human lysozyme that contains an internal targeting signal which directs the recombinant protein to the PBs [62]. Clearly, further studies are required to confirm targeting of the BP178 peptide to PBs and to identify the molecular determinants involved.

Fractionation procedures, in combination with western-blot and LC-MS/MS analyses allowed us to demonstrate that the BP178 peptide was efficiently produced and accumulated in the endosperm in BP178-transgenic events. In addition, this production was without apparent interference on the accumulation of the seed storage proteins such as glutelins and prolamins. In contrast, other studies reported adverse effects of peptide accumulation in rice seeds. For instance, the production of transgenic rice seeds accumulating the major Japanese cedar pollen allergens, Cry j 1 and Cry j 2 at high levels, was accompanied by an important reduction in the accumulation of endogenous seed storage proteins [63]. Similarly, a high level of accumulation in the transgenic seeds of a hybrid peptide comprising seven predominant human T cell epitopes resulted in alterations in the accumulation of seed storage proteins [64]. Presumably, the accumulation of the BP178 peptide in the endosperm of the rice seed prevents the ER stress response as previously described by other authors [65]. Alternatively, the accumulation of the BP178 might not have reached the threshold level required to induce ER stress.

The *in planta* produced BP178 peptide exhibited an apparent molecular weight in Tris-Tricine SDS-PAGE (mobility) higher than expected (~8 kDa compared to 3.2 kDa for this synthetic peptide). A similar phenomenon has been reported in transgenic plants expressing other AMPs [54, 55, 60, 66]. Different possibilities can explain the abnormal electrophoretic mobility of the plant produced BP178 peptide, such as the formation of self-assembled peptide multimers, an additional amino acidic fragment between the C-terminal of the signal peptide sequence (SGA---) and the N-terminal sequence of the BP178 peptide (---KKL), post-translational modifications or an improper processing of the signal peptide. However, the tryptic peptide corresponding to the signal peptide was not detected by LC-MS/MS analysis of the seed produced BP178 peptide. It is also known that proteins that enter into the secretory pathway are often modified in plant cells by the addition of sugar chains. Probably, the strong basic and amphipathic character of the BP178 and/or multimerization might be responsible of its abnormal electrophoretic mobility in SDS-PAGE gels.

A successful use of rice seeds as biofactories of AMPs requires the stable expression of the transgene through successive generations of plants, as well as stability of the peptide during seed storage. BP178 accumulation remained stable during storage of the rice seeds, at least for three years, without significant loss of antimicrobial activity. This stability can be associated to the adequate biochemical environment of the PBs, which might protect the peptide from the action of endogenous proteases [27, 29, 31, 54]. Furthermore, the amino acid composition and structure of the BP178 peptide can be also responsible for the integrity of the peptide under these conditions as has been argued for other peptides [53].

In the present work, a simplified method to recover BP178 peptide from seeds was developed. The procedure consists of a two-step centrifugation to partially-purify protein bodies, from which the peptide was solubilized in an acetone mix, whereas the seed storage proteins were precipitated and removed. The plant derived BP178 peptide showed bactericidal activity. Although the activity was similar to the synthetic BP178 peptide, the concentrations were around forty times higher. It is possible there was a loss of activity of the plant derived BP178 peptide related to its abnormal electrophoretic mobility.

In the literature there are many examples of AMP genes that have been successfully expressed in rice to obtain disease resistant transgenic plants [56, 66–71]. However, less attention has been paid to develop plant biofactories for the production of AMPs and to recover the expected product. The processes for extraction and purification of peptides in general and of cationic amphipathic AMPs in particular, are often laborious and costly, often requiring several steps [42, 72], and frequently complicated by the interaction of the expression product with tissue components or with the equipment used [40, 54, 73].

Current literature on production of peptides in rice seeds shows both successful and unsuccessful experiences, but in most cases repeated tandems or fusion proteins were used [52, 74–76]. The system here developed for production of BP178 was efficient without the need of using repeated tandems or fusion of the AMP to a larger protein carrier.

Among the three strategies assayed in this work (e.g. promoter used to drive transgen expression), seeds from *pGlb1:BP178* rice plants showed the highest accumulation levels of BP178 in the seed endosperm (up to 21 μg/g of seed, as revealed by western blot analysis and densitometric quantification). However, this value may represent an underestimation of the actual concentrations, because immunoblot detection of short and basic AMPs might underestimate the peptide yield [55, 66, 77]. In addition, the yield of BP178 (6.33 pmols/mg of seed) was in the upper range of the above mentioned reports (0.03 to 10 pmols/mg of seed) and four times higher than for cecropin A using a similar strategy (1.5 pmols/mg of seed) [54]. The observation that the *in planta* produced BP178 peptide was biologically active and effectively protects germinating seeds against phytopathogens is in agreement with the accumulation of effective levels to confer antimicrobial activity [52]. This data additionally shows that the production of BP178 appears as a useful approach for engineering broad-spectrum protection against seed-borne pathogens in rice grains.

In summary, the strategy presented here for expression of peptide BP178 solves the limitation that was encountered with genes encoding highly cationic α-helical small peptides showing toxicity towards the host plant [55, 56]. Overall, these results suggest that the endosperm tissue of the rice seed can be considered as an appropriated platform for the accumulation of BP178, with no need of complex downstream processing steps for the elimination of endogenous storage proteins. The method requires minimum purification and processing steps, due to the solubility in acetone of the transgene product, with a yield suitable for their purification and further use of BP178 as an antimicrobial agent. The system also allows for long-term storage of the AMP-accumulating rice seeds and does not affect the normal growth and development of the plant. Thus, the rice-based BP178 peptide plant biofactory offers new possibilities for the sustainable production of this peptide and application, not only in crop protection, but also in other fields (pharmaceuticals, preservatives).

## Conclusions

The strategy presented here for expression of peptide BP178 solve the limitation that was encountered with genes encoding highly cationic α-helical small peptides showing toxicity towards the host plant. Overall, these results suggest that the endosperm tissue of the rice seed can be considered as an appropriated platform for the accumulation of BP178, with no need of complex downstream processing steps for the elimination of endogenous storage proteins. The method requires minimum purification and processing steps, due to the solubility in acetone of the transgene product, with a yield suitable for their purification and further use of BP178 as an antimicrobial agent. The system also allows for long-term storage of the AMP-accumulating rice seeds and does not affect the normal growth and development of the plant. Thus, the rice-based BP178 peptide plant biofactory offers new possibilities for the sustainable production of this peptide and application, not only in crop protection, but also in other fields (pharmaceuticals, preservatives).

## Methods

### Construction of vectors for rice transformation

Synthetic *BP178* gene was designed based on the codon usage bias in *Oryza sativa*, chemically synthetized and cloned into the pUC57 plasmid (GenScript).

Three constructs for the expression of the *BP178* gene in rice plants were prepared (Fig. 1).

To drive expression of the *BP178* gene, three rice seed endosperm-specific promoters were chosen: *glutelin B-1* (*GluB1*), *glutelin B-4* (*GluB4*) and *26 kDa* α-*globulin* (*Glb1*) [35]. Additionally, the signal peptide sequence of the corresponding rice endosperm storage protein (GluB1, GluB4 or 26 kDa Glb1) was fused to the N-terminus of the BP178 peptide sequence to direct its internalization into the ER system of the plant cell, and presented in Additional file 1: Figure S1. The sequence encoding the KDEL tetrapeptide (ER-retention signal) was added in C-terminal.

The various endosperm-specific promoters containing the 5′ untranslated region were isolated from genomic DNA obtained from rice leaves (*Oryza sativa* ssp. *japonica* cv. Senia, acquired from the rice source of the Centre for Research in Agricultural Genomics, Barcelona, Spain). Oligonucleotide primers designed according to the nucleotide sequences available at the EMBL/GenBank/DDBJ nucleotide sequence database with accession numbers AY427569 (*GluB1*), AY427571 (*GluB4*) and AY427575 (*Glb1*) [35] for the Nipponbare *japonica* rice cultivar are shown in Additional file 5. The tissue-specificity and activity of the three rice seed promoters used in this work was previously described [35]. The *26 kDa globulin* promoter was found to be active in the inner starchy endosperm tissue, whilst the glutelin promoters exhibited the highest promoter activity in the outer portion of the endosperm.

The nucleotide sequence for the *nopaline synthase* terminator (*nos*) from *Agrobacterium tumefaciens* was also obtained by PCR using the plasmid *pC::pUbi:Cec-AKDEL:nos* as the template [66]. All the PCR products were gel-purified using a DNA purification column and cloned into the pGEM ®T-Easy plasmid. Briefly, the *pGEM::nos* and *pGEM:endosperm promoter-signal peptide* (*SP*) vectors were digested with the appropriate restriction enzymes. The *Nos* terminator sequence was cloned downstream of the *promoter-SP*, resulting in plasmids *pGEM:endosperm promoter-SP:nos*. Subsequently, the synthetic *BP178* gene was cloned into the *pGEM::endosperm promoter-SP:nos* plasmid DNA using the indicated restriction enzymes (Fig. 1). Previously, restriction sites were introduced by PCR using the primers described in Additional file 6. After verification by sequencing, the complete cassette (*endosperm promoter-SP:BP178:nos*) was cloned into the pCAMBIA1300 plant expression vector, which already contains the *hptII* (*hygromycin phosphotransferase*) gene encoding hygromycin resistance in the T-DNA region.

The resulting binary vectors were transferred to *Agrobacterium tumefaciens* EHA105 (obtained from the Centre for Research in Agricultural Genomics, Barcelona, Spain) [78] for the stable rice transformation.

### Production and characterization of transgenic rice plants expressing the *BP178* gene in seed tissues

Transgenic rice plants were produced by *Agrobacterium*-mediated transformation [79] using the commercial cultivars Senia and Ariete (obtained from the rice source of the Centre for Research in Agricultural Genomics, Barcelona, Spain). Transformation with the pCAMBIA1300 empty vector was done in parallel as a control.

Transgene integration into the rice genome, and integrity was examined by PCR analysis using genomic DNA obtained from young leaves (T0 plants) [80]. PCR primers were designed to amplify either the *BP178* gene or the complete cassette for expression of the *AMP* gene (that is the *promoter:BP178:nos DNA* fragment). The transgene copy number was estimated in all T0 regenerated plants (and T3 plants) by quantitative PCR (qPCR) targeting to either the *AMP* or the *hptII* genes (primers, Additional file 7). The single copy gene, *β-actin*, was used for normalization as previously described [54, 56, 81].

To obtain homozygous lines and to analyse the *hptII* inheritance, 24 randomly chosen T1 seeds of the selected T0 transgenic lines were germinated in the presence of hygromycin. The offspring homozygous for *hptII* gene was identified in the T2 lines, and T3 lines were obtained by self-pollination of T2 plants. The primary transgenic and next seed progenies were soil-grown to maturity in the greenhouse at 28 °C/20 °C (day/night) using a 12 h/12 h (day/night) photoperiod.

### Purification and detection of the BP178 peptide from protein bodies of rice seeds

A simplified method for preparation of partially purified protein bodies was previously reported [54] and used in the present work. Briefly, 1500 (30 g of seed) dehulled mature seeds were soaked in water for 2 h and were homogenized at 4 °C in 100 mL of grinding buffer (0.6 M Sucrose, 10 mM Na_2_HPO_4_, pH 7.5) and 7 mL of enriched protein body fraction were obtained. Protein extracts (50 μg) from PB enriched fractions were analysed on a Tris-Tricine SDS-PAGE gel, blotted onto 0.2 μm PVDF membrane and probed with the antibody raised against the BP178 peptide (1:2000, GenScript). Binding was detected using an Anti-Rabbit IgG (Fc) Alkaline Phosphatase conjugate secondary antibody (1:7500, Promega). The Western Blue®Stabilized substrate served for visualization of alkaline phosphatase activity (Promega). The signal of the BP178 peptide accumulated in the PB of transgenic lines was quantified using the Quantity Tools Image Lab™ Software (Version 4.1) included in the ChemiDoc™ XRS+ System (Bio-Rad, USA), by comparing the band intensity of the BP178 produced in seeds to that of reference amounts of synthetic BP178 peptide at known concentrations, run in the same gel. Since synthetic peptide shows generally n-mer bands in western-blot analysis, the amount of the standard peptide was calculated taking into account the sum of all individual immunodetected bands. BP178 peptide was synthesized by the LIPPSO Laboratory (Innovation Laboratory in Organic Chemistry Processes and Products, University of Girona, Spain).

Crude protein from protein body enriched fraction was obtained by adding ice cold-acetone (acetone-TCA-DTT) containing 15% (*v/v*) TCA and 20 mM DTT to the pelleted protein bodies, incubation for 60 min at −20 °C and centrifugation at 13,000 × rpm for 23 min at 4 °C. After centrifugation, the supernatant was recovered and evaporated to finally obtain a solid fraction enriched with BP178 peptide free of starch, cell debris and most of native seed storage proteins. Both, the supernatant and pellet were analysed.

The identification of the BP178 peptide in PB enriched fractions from transgenic rice seeds was performed using an Agilent 1200 Nanoflow HPLC coupled to a 4000 QTRAP LC-MS/MS hybrid triple quadruple/linear ion trap mass spectrometer using a microSpray source (AB/MDS Sciex) as described previously [54].

For the BP178 peptide, four transitions were selected, according to their relative intensity and m/z greater than the precursor m/z (Additional file 8).

### Antibacterial activity of the *in planta* produced BP178

The antibacterial activity of BP178 peptide produced in rice seeds was assessed in vitro against *Dickeya* sp. 1552 10.1 (provided by Maria López, Reference Laboratory in Plant Pathogenic Bacteria, Instituto Valenciano de Investigaciones Agrarias, Valencia, Spain) and compared to that of synthetic BP178. Two batches of 25 mg of the enriched protein body fraction (Additional file 5), were used to obtain a partially purified plant derived BP178, according to the procedure indicated in Fig. 4a, and then the acetone supernatant was evaporated. The dried supernatant from the acetone-TCA-DTT treated protein bodies was used. Because TCA inhibited bacterial growth, it was removed from the dried extract using three consecutive washing cycles with 10 mM acetic acid and evaporation. Finally, the dried BP178 acetate was solubilized in diethyl ether, evaporated, and resuspended in 20 μL water for the antibacterial assay. Western analysis was used to determine the BP178 contents were in each batch. Empty vector PB enriched extracts were treated similarly than BP178-seeds and included as control. Also synthetic BP178 (3.2 μM) followed the same steps of the above mentioned protocol used to obtain BP178 from seeds. For the assay, 10 μL of the bacterial cells suspension (2 × 10^4^ cfu/mL) were mixed with 10 μL of the 3.2 μM synthetic BP178 or of the purified BP178 from transgenic seeds, and incubated for 2 h at 28 °C.

All samples were prepared in duplicate. After incubation, aliquots of 10 μL were plated onto LB agar and grown for 24 h at 28 °C, and viable cells determined. The experiment was repeated twice.

### Resistance of BP178 rice seedlings to pathogen infection

A seed germination test was used to determine the resistance of BP178-seedlings to two plant pathogens, the bacterial pathogen *Dickeya* sp. and the seed-borne fungal pathogen *Fusarium verticillioides* (supplied by the Plant Health Services, Generalitat de Catalunya, Barcelona, Spain)*.* For the bacterial pathogen the assay was performed as previously described with minor modifications [56]. Twelve surface-sterilized seeds were placed in 24-well culture chambers and inoculated with 500 μL of water or a bacterial suspension (10^7^ cfu/mL) and vacuum infiltrated. For the fungal pathogen the procedure was as previously reported [54]. Seeds were inoculated with 50 μl of a spore suspension at a concentration of 10^3^ (cv. Senia) or 10^5^ (cv. Ariete) spores/ml (water as germination control) on MS medium without sucrose. In both cases seeds were allowed to germinate at 28 °C for seven days, using a 12 h/12 h (day/night) photoperiod under a photon flux of 110–150 μmol. m^−2^. s^−1^. Then, disease intensity was estimated using a semi-quantitative scale (0, not germinated seed; 0.5, germinated seed with the seedling length less than 25% of control; 1.0, germinated seed with the seedling length from 25 to less than 50% of control; 1.5, germinated seed with the seedling length from 50 to less than 75% of control; and 2.0, germinated seed with the seedling of the same length as the control seeds). Transgenic seeds were compared to WT and empty vector seeds, and to non-pathogen inoculated seeds.

## Additional files

Additional file 1: Nucleotide sequences of the synthetic *BP178* gene and the corresponding translated amino acid sequences. (TIF 125 kb)
Additional file 2: Antibacterial, hemolytic and phytotoxic activity of antimicrobial peptides of interest in this study. (XLSX 12 kb)
Additional file 3: Transgene integration in the rice genome of the generated transgenic lines. (TIF 264 kb)
Additional file 4: Preparation of the PB fraction from rice seeds. (TIF 1091 kb)
Additional file 5: Accumulation of BP178 in seed protein body enriched fractions of *pGlb1:BP178* transgenic rice lines (T1 generation). (TIF 184 kb)
Additional file 6: Oligonucleotides used to amplify the rice seed promoter and its signal peptide coding region, the *BP178* gene and the *nopaline synthase* terminator. (XLS 37 kb)
Additional file 7: Oligonucleotides used for quantitative polymerase chain reaction. (XLSX 11 kb)
Additional file 8: Selected transitions and parameters used in MS analysis of the BP178 peptide produced in rice seeds. (XLSX 10 kb)

## Abbreviations

°C: Degrees celsius
Aa: Amino acid
Ag: Antigen
AMP: Antimicrobial peptide
bp: Base pair
CaMV: *Cauliflower mosaic virus*
CE: Collision energy
Cec A: Cecropin A peptide
cfu/mL: Colony-forming units per millilitre
DP: Declustering potential
ER: Endoplasmic reticulum
EV: Empty vector
F: Forward
Glb-1: 26 kDa α-globulin
GluB-1: Glutelin B-1
GluB-4: Glutelin B-4
GUS: β-Glucuronidase
h: Hour
HPLC: High Performance Liquid Chromatography
*hpt II*: Selection gene encoding resistance to hygromycin
KDEL: ER retention signal sequence (aa sequence)
LB: Left border
LB broth, agar: Luria-Bertani
LC-MS: Liquid Chromatography-Mass Spectrometry
m/z: Mass divided by charge
mg: Milligram
MIC: Minimal inhibitory concentration
min: Minutes
mL: Millilitre
mM: Millimolar
Mw: Molecular weight
ng: Nanogram
*nos*: *Nopaline synthase terminator*
PB: Protein body
*pGlb-1*: *Glb-1 promoter*
*pGluB-1*: *GluB-1 promoter*
*pGluB-4*: *GluB-4 promoter*
pI: Isoelectric point
prom: Promoter
*pUbi*: *Ubiquitin promoter*
PVDF: Polyvinylidene difluoride
qPCR: Quantitative PCR
R: Reverse
RB: Right border
RS: Restriction site
SDS-PAGE: Sodium dodecyl sulphate-polyacrylamide gel electrophoresis
TCA: Trichloroacetic acid
term: Terminator
Tris: Tris (hydroxymethyl) aminomethane
WT: Wild-type
μg: Microgram
μL: Microlitre
μM: Micromolar
μm: Micrometre
μmol: Micromol
