# Supplementary figures and images for: Production of BP178, a derivative of the synthetic antibacterial peptide BP100, in the rice seed endosperm
Source: BMC Plant Biol. 2017 Mar 14;17:63. doi: 10.1186/s12870-017-1011-9 (PMC5351061; doi:10.1186/s12870-017-1011-9)

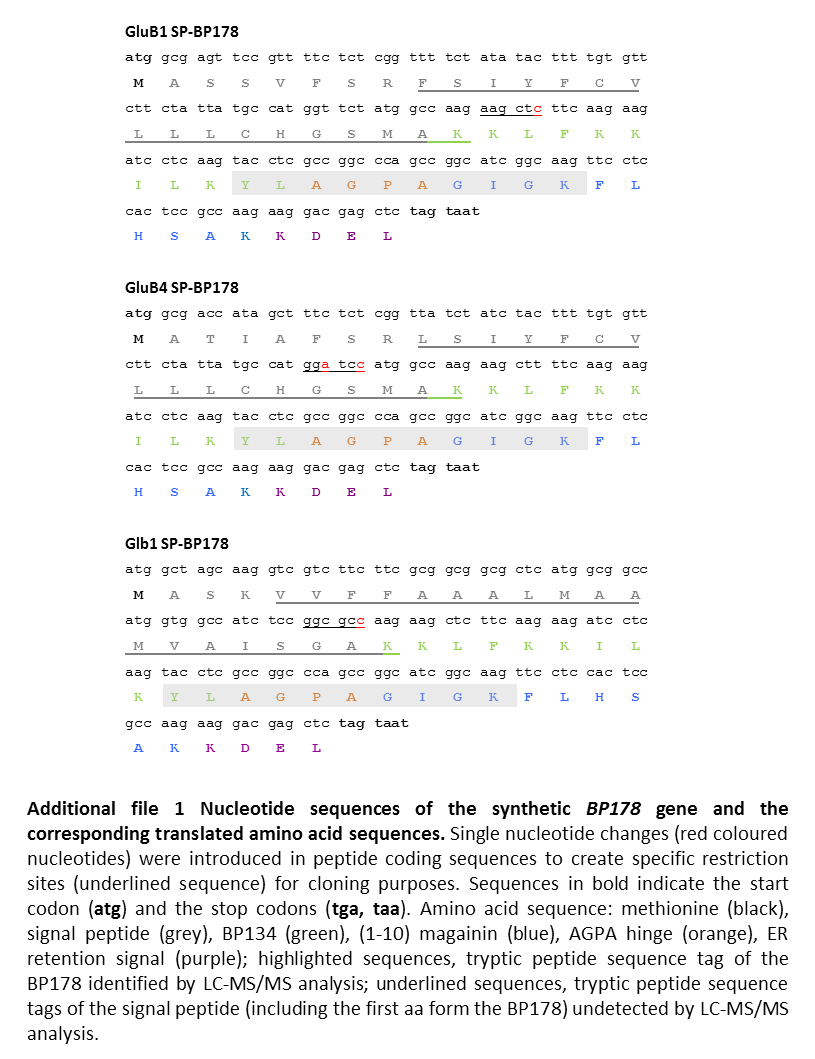

Supplement: Additional file 1: — Nucleotide sequences of the synthetic BP178 gene and the corresponding translated amino acid sequences. (TIF 125 kb) [file 12870_2017_1011_MOESM1_ESM.tif]

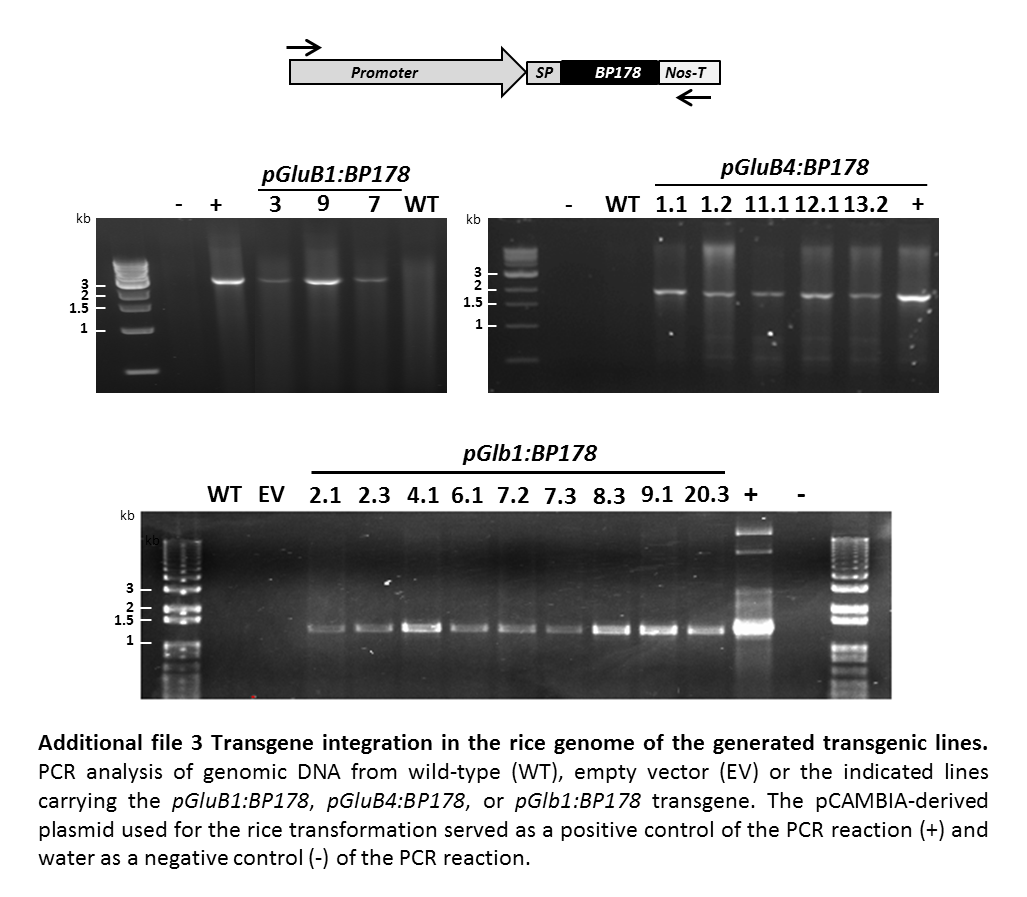

Supplement: Additional file 3: — Transgene integration in the rice genome of the generated transgenic lines. (TIF 264 kb) [file 12870_2017_1011_MOESM3_ESM.tif]

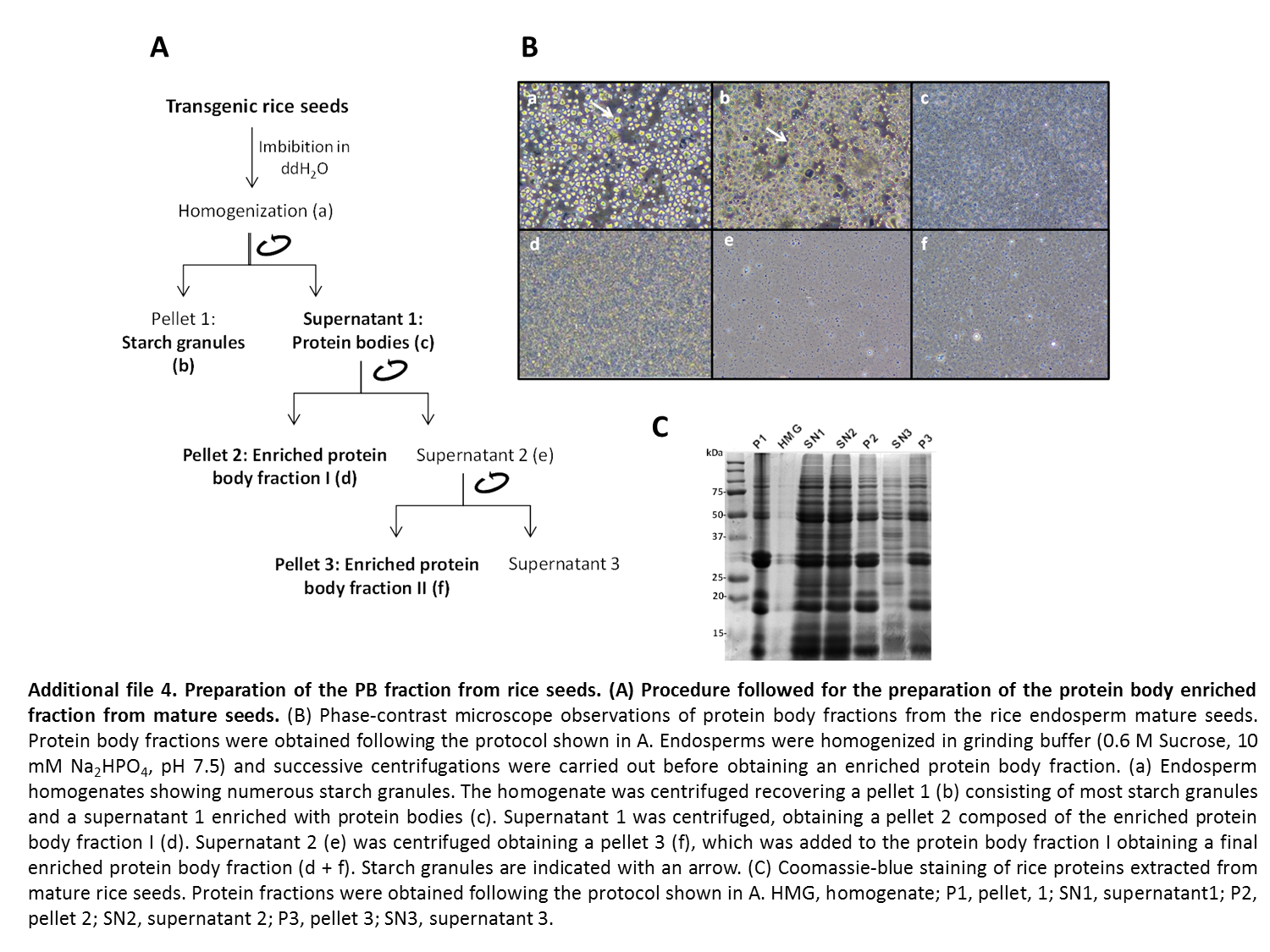

Supplement: Additional file 4: — Preparation of the PB fraction from rice seeds. (TIF 1091 kb) [file 12870_2017_1011_MOESM4_ESM.tif]

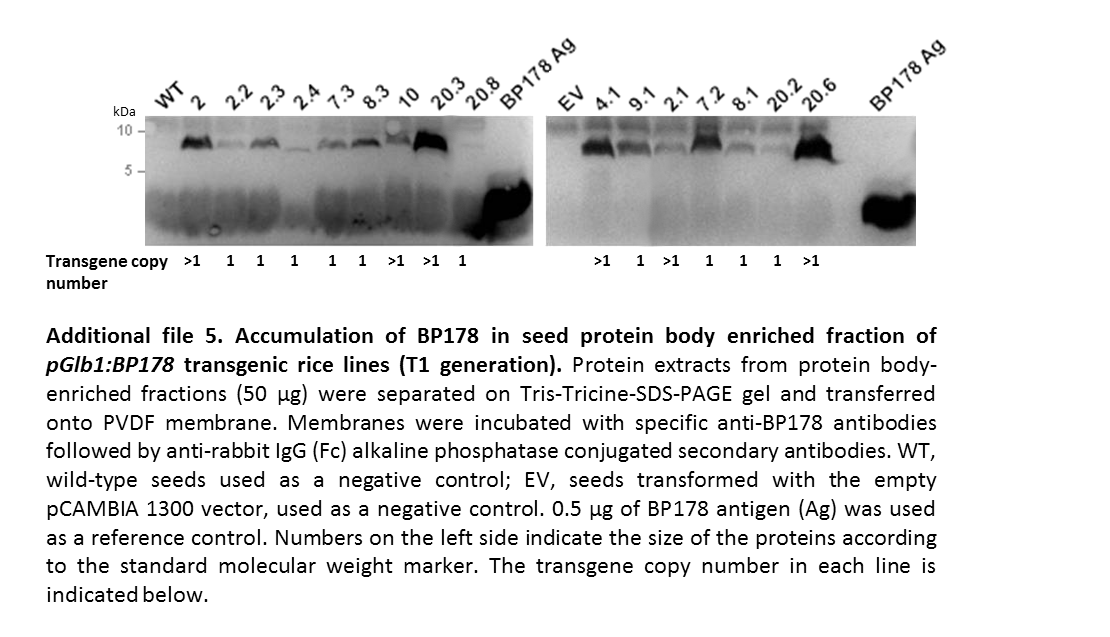

Supplement: Additional file 5: — Accumulation of BP178 in seed protein body enriched fractions of pGlb1:BP178 transgenic rice lines (T1 generation). (TIF 184 kb) [file 12870_2017_1011_MOESM5_ESM.tif]
